# 1951. Optimizing implementation of artificial intelligence-informed digital X-ray screening for tuberculosis through an age- and sex-differentiated approach

**DOI:** 10.1093/ofid/ofad500.105

**Published:** 2023-11-27

**Authors:** Joowhan Sung, Peter J Kitonsa, David Isooba, Susan Birabwa, Akash Malhotra, Annet Nalutaaya, David W Dowdy, Achilles Katamba, Emily A Kendall

**Affiliations:** Johns Hopkins University School of Medicine, Baltimore, MD; Uganda Tuberculosis Implementation Research Consortium, Kampala, Kampala, Uganda; Uganda Tuberculosis Implementation Research Consortium, Kampala, Kampala, Uganda; Uganda Tuberculosis Implementation Research Consortium, Kampala, Kampala, Uganda; Johns Hopkins Bloomberg School of Public Health, Baltimore, Maryland; Uganda Tuberculosis Implementation Research Consortium, Kampala, Kampala, Uganda; Johns Hopkins Bloomberg School of Public Health, Baltimore, Maryland; Makerere University College of Health Science, Kampala, Kampala, Uganda; Johns Hopkins University School of Medicine, Baltimore, MD

## Abstract

**Background:**

Computer-aided detection (CAD) using artificial intelligence (AI) to analyze chest radiographs is an important tool for community tuberculosis (TB) screening in high burden countries. Most current algorithms use a universal cut-off score to select individuals for confirmatory TB testing; however, using a tailored cut-off based on client demographics (age and sex) may improve performance.

**Methods:**

Community-based TB screening was conducted using portable X-ray with CAD (qXR, [Qure.ai, India]) as part of a cluster-randomized trial in Uganda. Individuals scoring above a specified threshold were offered sputum Xpert Ultra testing. This threshold was initially set at 0.5 (range: 0-1) but was later lowered to 0.2 and then 0.1 for research purposes. For clients with scores ≥ 0.1 who did not undergo Xpert testing, we used multiple imputation to infer Xpert-positive status. We assumed that those with X-ray scores < 0.1 would be Xpert-negative if tested, but considered 0.1% or 0.5% prevalence of Xpert-positive TB in sensitivity analysis. We compared the sensitivity and specificity of using a universal screening threshold of 0.5 versus tailored thresholds based on client age and sex.

**Results:**

A total of 15,375 individuals were screened using AI-interpreted digital X-ray, of whom 1,748 (11.4%) had valid sputum Ultra results; 157 (9.0%) tested positive. Assuming that people with qXR scores < 0.1 would test negative on sputum Ultra, the manufacturer-recommended universal threshold of ≥ 0.5 had an estimated sensitivity of 75.8% (95%CI: 70.3-81.6) and specificity of 95.3% (95% CI 95.2-95.4). A sex-differentiated threshold (≥ 0.35 for men, ≥ 0.8 for women) had similar specificity but significantly higher sensitivity: 81.3% (95% CI 76.3-86.3). Sex-stratified thresholds also outperformed a universal threshold when assuming higher positivity among people with qXR scores < 0.1. Stratification by age did not significantly improve accuracy.

**Table 1:**
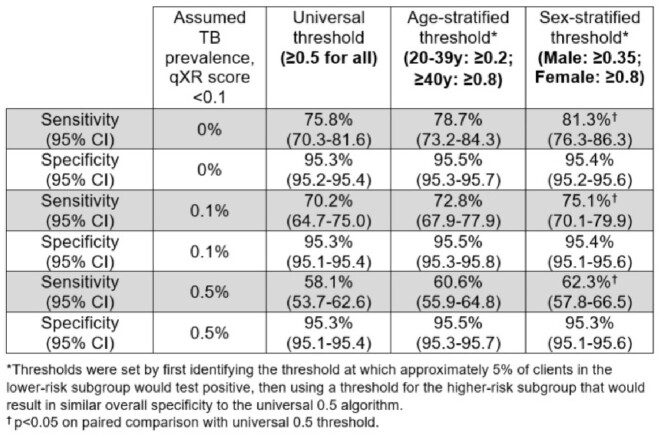
Sensitivity and specificity of different thresholds on computer-aided detection software (qXR) when using digital chest X-ray for detecting pulmonary tuberculosis.

**Figure 1.**
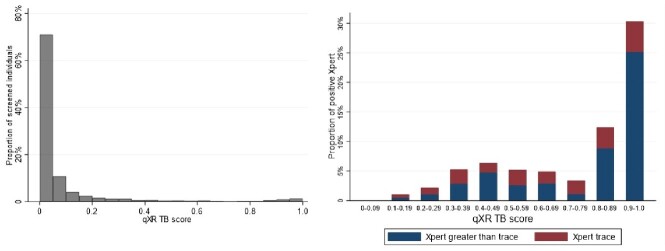
Distribution of CAD scores among individuals screened for TB using digital chest radiograph in Uganda (left) and the proportions of individuals with positive and trace sputum Xpert MTB/RIF Ultra results according to CAD score (right).

**Figure 2.**
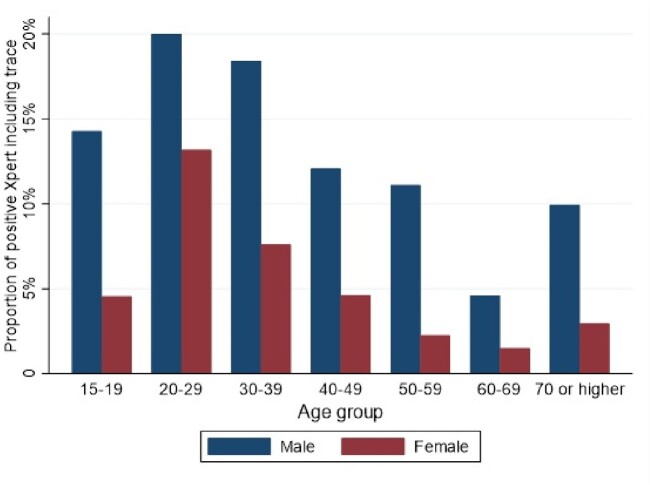
Proportion of individuals with positive sputum Xpert Ultra (including trace) according to age and sex.

**Conclusion:**

Using differentiated CAD score thresholds based on client sex could improve the accuracy of digital X-ray for TB screening. Future research should validate these findings in other populations and explore the value of incorporating additional client characteristics into TB screening algorithms.

**Disclosures:**

**All Authors**: No reported disclosures

